# How do the strength and type of ENSO affect SST predictability in coupled models

**DOI:** 10.1038/srep33790

**Published:** 2016-09-21

**Authors:** Soo-Jin Sohn, Chi-Yung Tam, Hye-In Jeong

**Affiliations:** 1 Climate Prediction Department, APEC Climate Center (APCC), Busan, Republic of Korea; 2 Earth System Science Programme, The Chinese University of Hong Kong, Hong Kong, China

## Abstract

The effects of amplitude and type of the El Niño-Southern Oscillation (ENSO) on sea surface temperature (SST) predictability on a global scale were investigated, by examining historical climate forecasts for the period 1982–2006 from air-sea coupled seasonal prediction systems. Unlike in previous studies, SST predictability was evaluated in different phases of ENSO and for episodes with various strengths. Our results reveal that the seasonal mean Niño 3.4 index is well predicted in a multi-model ensemble (MME), even for four-month lead predictions. However, coupled models have particularly low skill in predicting the global SST pattern during weak ENSO events. During weak El Niño events, which are also El Niño Modoki in this period, a number of models fail to reproduce the associated tri-pole SST pattern over the tropical Pacific. During weak La Niña periods, SST signals in the MME tend to be less persistent than observations. Therefore, a good ENSO forecast does not guarantee a good SST prediction from a global perspective. The strength and type of ENSO need to be considered when inferring global SST and other climate impacts from model-predicted ENSO information.

The El Niño-Southern Oscillation (ENSO) is the most dominant forcing factor of inter-annual climate variability^1^,^2^,^3^. ENSO can greatly influence the climate in the tropics as well as more remote regions, including East Asia and even the mid-latitudes^4^,^5^. Its phase and amplitude can provide a foundation for regional climate forecasts^6^. Many seasonal prediction schemes are constructed based on information about sea surface temperature (SST)^7^ due to its important role in inciting low-frequency atmospheric changes^1^,^8^. For example, skilful forecasts of the Indian summer monsoon rainfall can be obtained based on global SST evolution at long lead times^9^. Some studies have also reported that regional climate variability can be influenced by local SST changes^10^,^11^. It is clear that SST is one of the key parameters when considering seasonal to inter-annual climate variations^12^.

Because of their importance, the ENSO phenomenon and global SST changes are constantly monitored by most operational centres. Some centres, including the Japan Meteorological Administration (JMA), National Centers for Environmental Prediction (NCEP), Korean Meteorological Administration (KMA), Australian Government Bureau of Meteorology (BoM), APEC Climate Center (APCC) and others, even provide forecasts of their evolution and the associated climate anomalies. Although numerous studies of the predictability of ENSO and its climate impacts have been conducted, the possible roles of the intensity and type of ENSO on SST predictions are still not well established. It has been found that a model’s skill at ENSO prediction is higher during El Niño/La Niña than in normal periods^13^. In fact, ENSO is better predicted during its growth than during its decay phase. It is also known that ENSO predictability has seasonal dependency by the phase locking of the ENSO to the annual cycle;^14^ for instance, its evolution is more difficult to predict in boreal spring (the so-called “spring predictability barrier”)^15^. Recently, a new type of ENSO has been discovered that differs from the canonical type. Known as the central-Pacific El Niño/El Niño Modoki, its warm phase is characterized by anomalous SST warming over the central Pacific, rather than over the far eastern Pacific^16^,^17^. The climate impacts of ENSO Modoki are also decidedly different from those of typical canonical ENSO events^18^,^19^,^20^,^21^,^22^. However, relatively few studies have addressed the predictability of ENSO, and SST predictability in general, based on the strength and type of ENSO using coupled general circulation models (CGCM)^23^,^24^. Hence, it is very important to consider how seasonal climate predictability might depend on the occurrence of different types of ENSO.

The aim of this study is to assess the skill of CGCM-based seasonal forecast systems at predicting ENSO occurrences and their associated SST changes for all seasons using the APCC multi-model ensemble (MME). ENSO events were identified based on the Niño 3.4 index, which is defined as the area-averaged anomalous SST within 5°S–5°N, 170°–120°W. The index is considered a key indicator of ENSO by most operational centres (e.g., NCEP, KMA, BoM, and North American Multi-Model Ensemble (NMME)). An El Niño is defined when the 5–month running mean of this index exceeds 0.4 °C for at least six months^25^. The Oceanic Niño Index (ONI), constructed based on SST over the Niño 3.4 region, is also adopted by the US Climate Prediction Center (CPC), and is used by the World Meteorological Organization (WMO) for monitoring ENSO and assessing its regional climate impacts^6^. We particularly focused on SST predictability in CGCMs and its relationship to the type and strength of ENSO.

## Results

**Figure 1:**
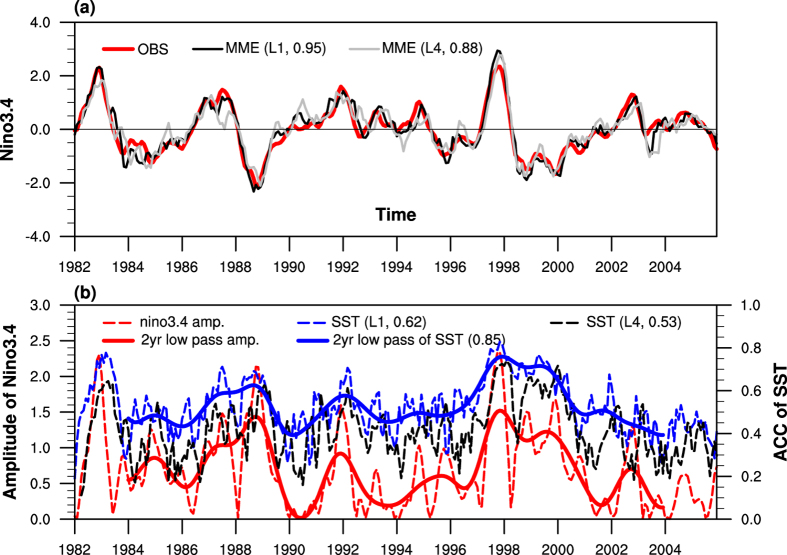
(**a**) Time series of the monthly mean Niño 3.4 index from observations (red line), one-month (black) and four-month (grey) lead MME simulations with year-round 3-month running average taken, within the period of 1982 to 2006. Correlation coefficients between the observed and MME simulated indices are given in parentheses following the legends. (**b**) Time series of the observed amplitude (absolute value of Fig. 1a) of Niño 3.4 (red dashed line) and anomaly pattern correlation (ACC) skill of global SST (60°S–80°N, 0°–330°E) prediction for one-month (blue dashed line) and four-month lead (black dashed line). Solid lines show the 2-year low pass filtered data of the Niño 3.4 amplitude and one-month global SST prediction skill time series. These figures are generated by the NCAR Command Language (Version 6.3.0) [Software]. (2016). Boulder, Colorado: UCAR/NCAR/CISL/TDD. http://dx.doi.org/10.5065/D6WD3XH5.

First, we considered ENSO predictability based on MME by comparing the observed Niño 3.4 index with the ones derived from one-month and four-month lead MME mean forecasts. Figure 1a shows the 3-month running mean results for all seasons. Overall, both one- and four- month lead MME predictions are skilful in capturing SST variability over the Niño 3.4 region, with the former (latter) giving a temporal correlation of 0.95 (0.88) with observations. On the other hand, there are periods in which model results deviate considerably from the observed Niño 3.4 index, especially for the four-month lead MME forecasts. This can be seen during 1984–1986 when Niño 3.4 SST was colder than normal, and also over the extended period from 1990 to 1996 when anomalously warm SST tended to persist. Interestingly, the SST signals were not strong in both epochs. The above thus hints at a possible relationship between the strength of ENSO and SST predictability in models.

Figure 1b gives the global SST prediction skill from the MME, computed based on the pattern correlation between hindcasted and observed SST, as well as the absolute magnitude of the observed Niño 3.4 index. From 1982 to 2006, the one-month lead SST forecast skill (denoted by blue dashed line) is significantly correlated with the strength of ENSO (with correlation of 0.62). High skills were found in the 1982/1983, 1987/1988 and 1997/1998 periods when El Niño was strong. Removing the biennial signals related to ENSO phase locking and the seasonality of forecast skill^26^, these time series made clear that a model’s skill was strongly correlated with the ENSO amplitude (with correlation of 0.85). When the amplitude of the Niño 3.4 index was large, the MME gave better predictions of the SST pattern over the whole globe. On the other hand, the skill score of global SST forecasts tended to be weaker during the epochs of 1984–1986, the early to mid 1990s, and from 2001 to 2005, when the ENSO signal was weaker. The skill for four-month lead SST prediction is also shown in Fig. 1b (see dashed line in black). Overall it gives a lower value than the one-month lead prediction skill score. Similar to the one-month lead case, the four-month lead SST prediction skill is well associated with the ENSO strength (with correlation of 0.53). In summary, both the one- and four- month lead global SST predictions show limited skills during weak ENSO episodes.

**Figure 2:**
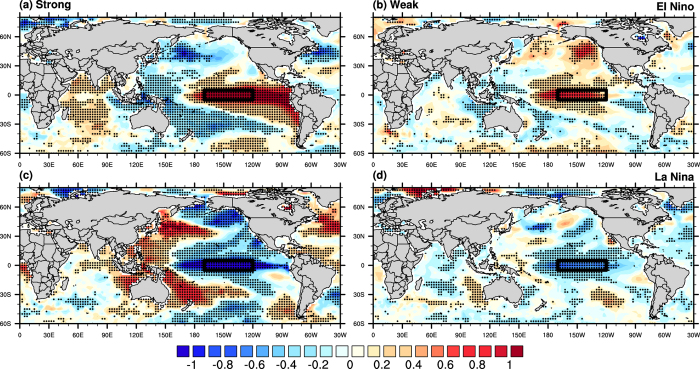
Composite maps of anomalous SST (shading) during (**a,b**) El Niño and (**c,d**) La Niña phases corresponding to (**a,c**) strong and (**b,d**) weak ENSO events for boreal warm season. Black dots indicate grid points where the anomalies are statistically significant at the 95% level. The Niño 3.4 region is denoted by a rectangular box in each panel. These figures are generated by the NCAR Command Language (Version 6.3.0) [Software]. (2016). Boulder, Colorado: UCAR/NCAR/CISL/TDD. http://dx.doi.org/10.5065/D6WD3XH5.

In view of the relationship between the ENSO amplitude and SST predictability, ENSO events were classified according to the strength of the Niño 3.4 index (see Methods). For the warm ENSO phase, three out of the four strong events correspond to canonical El Niño, whereas all four weak events could be classified as El Niño Modoki within the 1982–2006 period (see Tables S1 and S2). Figure 2 shows the corresponding SST composites in the boreal warm season. During a strong El Niño, positive anomalies were found within the deep tropical central-to-eastern Pacific, and negative signals over the western equatorial Pacific (see Fig. 2a). The SST is warmer than normal over the Niño 3.4 region; this SST pattern apparently matches that for canonical El Niño. In comparison, during weak El Niño events, SST was anomalously warm over the central equatorial Pacific, with cooler-than-normal SST to the west and east (Fig. 2b). The tri-pole SST pattern is reminiscent of that associated with El Niño Modoki. To quantify the degree of resemblance between these composites and those related to the two El Niño types, observed SST data was first regressed onto the Niño 3 index and El Niño Modoki index (EMI) (see Supplementary Fig. S2). The pattern correlation between the canonical El Niño (El Niño Modoki) SST regression map and the aforementioned strong (weak) El Niño SST composite was found to be 0.89 (0.71) (see also Supplementary Fig. S1 for similar results in the boreal cold season). It was apparent that a strong (weak) warm El Niño mainly corresponded to a canonical El Niño (El Niño Modoki).

In contrast to El Niño cases, it is noteworthy that only two out of five weak La Niña events corresponded to La Niña Modoki. During strong La Niña years, lower than normal ocean temperature was observed across the eastern-central Pacific. However, in weak La Niña periods, anomalous SST along the equatorial Pacific did not exhibit a tri-pole-like (i.e. warm–cold–warm) pattern. Also, notice the significantly warm SST in the north western Pacific during strong La Niña; for weak events SST signals were much weaker in the same region. The correlation between the strong (weak) La Niña SST pattern and the SST regression map based on the Niño 3 (El Niño Modoki) index was only −0.6 (−0.38). It is clear that a weak La Niña does not always correspond to a La Niña Modoki.

**Figure 3:**
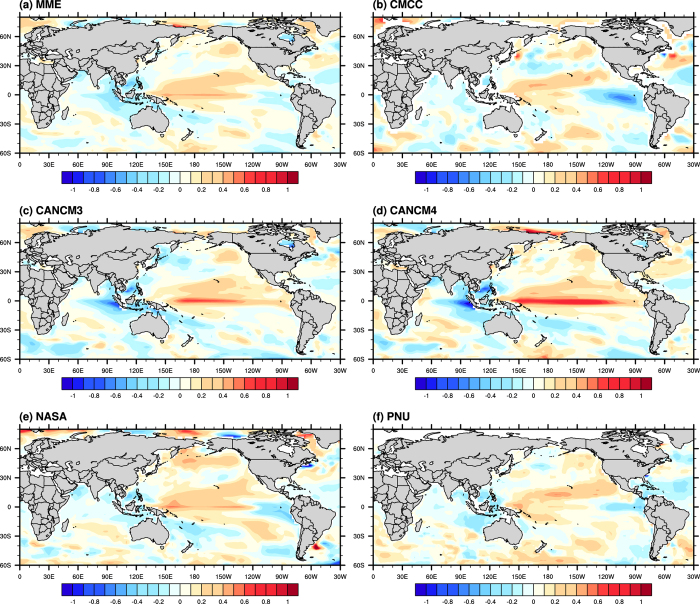
Composite maps of anomalous SST (shading) for weak El Niño events during the boreal warm season based on (**a**) MME mean and individual model simulations from (**b**) CMCC, (**c**) CANCM3, (**d**) CANCM4, (**e**) NASA and (**f**) PNU. These figures are generated by the NCAR Command Language (Version 6.3.0) [Software]. (2016). Boulder, Colorado: UCAR/NCAR/CISL/TDD. http://dx.doi.org/10.5065/D6WD3XH5.

Model simulated SST patterns for strong and weak ENSO events are now examined. In general, SST anomalies during strong ENSO events from all of the climate models considered compare well with their observational ones (see Figs S3 and S4). On the other hand, some models seem to have difficulty in reproducing SST patterns associated with weak ENSO events. Figure 3 gives the SST composites from individual models and their MME mean for weak El Niño events in the boreal warm season. During weak El Niño years, all models can capture the warm SST signals typically found near the dateline. As mentioned, such SST greatly resembles that during El Niño Modoki; on the other hand the anomalously cool regions in both western and eastern Pacific are not well captured by some models. In the cold season, models also tend to give weaker cold anomalies than observations in the eastern equatorial Pacific for weak El Niño events (see Fig. S5).

**Figure 4:**
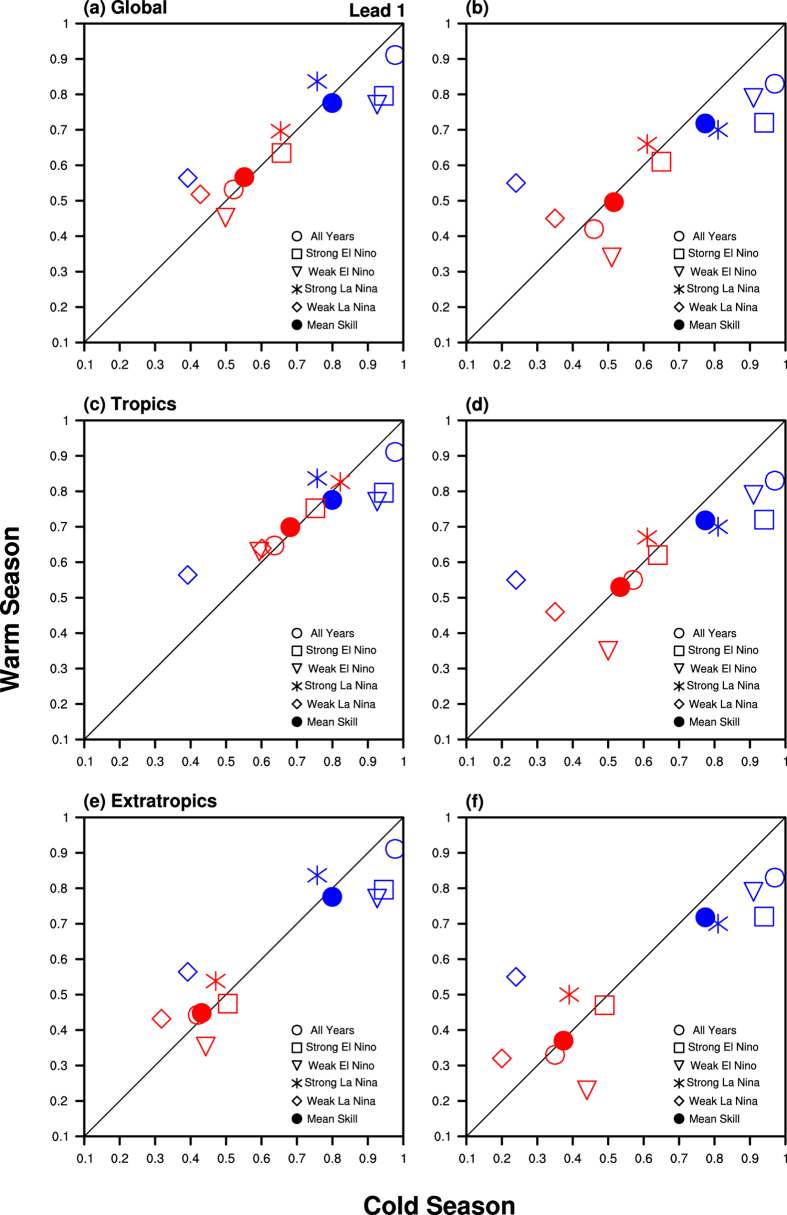
(**a,b**) Skills of Niño 3.4 index forecasts (in blue) and SST pattern forecasts (in red) over the globe (i.e. same area as for Fig. 1), during boreal cold (x axis) and warm (y axis) seasons for all years as well as for strong El Niño, weak El Niño, strong La Niña, and weak La Niña events (see legends in figures). (**c,d**) Same as (**a,b**) except for SST pattern forecasts over the tropics (30°S–30°N). (**e,f**) Same as (**a,b**) except for SST pattern forecasts over the extratropics (60–30°S, 30–80°N). Results are based on (**a,c,e**) one-month lead, and (**b,d,f**) August and November initiated MME mean predictions. The index forecast skill is defined as the correlation between the observed and simulated MME mean Niño 3.4. The SST pattern forecast skill is defined as the anomaly pattern correlation between SST from observations and MME simulations. These figures are generated by the NCAR Command Language (Version 6.3.0) [Software]. (2016). Boulder, Colorado: UCAR/NCAR/CISL/TDD. http://dx.doi.org/10.5065/D6WD3XH5.

To further investigate SST predictability and its relationship with the ENSO strength, the skills of the Niño 3.4 index and SST pattern forecasts for each categorised ENSO events and for both boreal summer and winter are compared (see Fig. 4). In general, coupled models have high fidelity in predicting the time variation of central-to-eastern tropical Pacific SST. Based on data for all years, it can be seen that SST forecast skills during warm and cold seasons are comparable (see solid dots in Fig. 4); there is also evidence that the Niño 3.4 index is better predicted in boreal winter than in summer during El Niño. The latter is likely related to the seasonal phase locking of ENSO predictability, with short-lead predictions having a relative low skill in boreal summer^27^. Consistent with our previous results, SST tends to be more predictable during strong than during weak ENSO events. Particularly low skill in predicting the Niño 3.4 index was found in weak La Niña years, with temporal correlation between MME and observations as low as 0.2; the same metric is ~0.7 to 0.8 during strong La Niña years. Inspection of SST maps indicated that model tends to give less persistent signals during 1985/1986, 1995/1996, and 2000/2001 (see Fig. S6). This might be the reason that the MME performed poorly in these weak La Niña years.

The strength of ENSO events is even more crucial in determining a model’s skill at capturing the accompanying global SST patterns. During strong El Niño, the global SST was well forecasted (with pattern correlation between model and observations up to 0.66). It should be mentioned that this was not true for weak El Niño (with pattern correlation of ~0.34 to 0.5). This is consistent with our previous results regarding strong and weak El Niño SST forecasts (see Fig. 3). Global SST is also well predicted when La Niña is strong (with pattern correlation ranging from ~0.6 to 0.7), but not for weak La Niña (pattern correlation ~0.35 to 0.5). Inspection of individual models revealed the same tendency, with a model’s skill in capturing global SST being higher (lower) for strong (weak) ENSO events (see Fig. S7). Moreover, it appears that such dependence of SST predictability on ENSO’s strength is independent of forecast lead time (see Fig. S8). We have repeated the same analysis by considering forecast skills for SST patterns in tropical and extratropical regions separately (see Fig. 4c–f). It can be seen that models tend to give better SST predictions in the tropics, compared to those over the extratropics. For instance, the SST pattern correlation from one-month lead forecasts can be 0.7 or more over the tropical zone, whereas outside the tropics it is only about 0.4 to 0.5 from the same set of forecasts. Again it is clear that coupled models have difficulties in predicting SST changes during weak ENSO, and this is true over both tropical and extratropical oceans.

## Discussion

Effects of the strength and type of ENSO on global SST predictability in the APCC MME seasonal forecast system were assessed based on hindcasts targeting all seasons for the period 1982–2006. In general, seasonal SST predictability depends strongly on the phase as well as the intensity of ENSO. The Niño 3.4 index is well predicted by coupled models. However, they tend to perform better in capturing variations of the Niño 3.4 index during strong ENSO events, than in periods when ENSO is weak in their long-lead (4-month lead) predictions. It is probably due to the fact that models’ ability in capturing ENSO evolution is mainly determined by ocean dynamics; strong events can lead to stronger anomalous states in the ocean component, which might be conducive to better ENSO simulations as the lead time increases. The global SST prediction skill is even more sensitive to the strength of ENSO. The strongest El Niño events identified in the hindcast period belong to canonical El Niño, whereas all weak events were found to correspond to El Niño Modoki. During those strong events, SST changes around the globe were well predicted by models. However, a number of models failed to reproduce the SST anomalies typically associated with an El Niño Modoki (especially those over the far eastern Pacific), resulting in relatively poor skill by the MME in capturing global SST variations in weak El Niño years. When La Niña is weak, models tend to give central-to-eastern Pacific SST signals, which are less persistent than observations, and global SST forecasts also perform poorly compared to those during strong La Niña cases. Because of this sensitivity of SST prediction skills to the type of ENSO, more studies are needed to determine the various mechanisms that trigger and sustain the two types of ENSO events, and to assess how well these mechanisms are reproduced in state-of-the-art coupled models. It has been suggested that a strong or canonical ENSO is related to tropical atmosphere–ocean coupling and tied to thermocline variations. On the other hand, weak El Niño or El Niño Modoki is related to the Pacific subtropical high and the Asian–Australian monsoon system, and could be more influenced by atmospheric forcing and local air-sea coupling rather than basin-wide ocean dynamics^28^. These different triggering mechanisms might also lead to differences in models’ different skills at predicting the two types of ENSO^29^. It is important to determine how well these regional climate signals can be assimilated by CGCM climate prediction systems.

Our results also highlight the problem of adopting a single index for ENSO prediction. Although it is the most widely used ENSO index, especially within the seasonal climate prediction community, the use of the Niño 3.4 index only might risk mixing the two types of ENSO. Using only SST over the central equatorial Pacific, and without taking into account conditions farther east, the Niño 3.4 index cannot distinguish between canonical and atypical El Niño events. The different types of El Niño are also known to produce different teleconnection patterns and hence very different climate impacts across the globe^6^. It is therefore imperative to differentiate signals related to the different ENSO types, so as to enable more accurate information to be used for regional climate assessments and seasonal forecasts.

## Methods

In this study, the optimum interpolation (OI) of SST data^30^ was used as observations for examining ENSO and its associated SST variability. To assess the ability of ocean–atmosphere coupled models^31^,^32^,^33^,^34^ to predict ENSO, seasonal hindcasts from five independent climate models contributing to the MME suite of APCC, Korea, were considered. Table 1 summarises these experiments and provides the model specifications, their ensemble sizes, and other relevant parameters. In all hindcast experiments, the models were initiated one month before the particular target season. Using these datasets, we have assessed one-month as well as four-month lead prediction skills for all consecutive three-month periods around the calendar year. In addition, SST predictions during the boreal warm season (defined as the average of JJA, JAS, ASO, and SON forecasts, with May 1 taken as the initial conditions) and cold season (defined as the average of DJF, JFM, and FMA forecasts, all initiated on November 1) were also examined. The MME mean is simply the average of the ensemble mean forecasts^35^ from the five CGCMs. All data cover the common hindcast period of 1982–2006 and all were first interpolated onto the same 2.5° × 2.5° grid prior to any comparison.

To determine the ENSO phase and amplitude, the 3-month running mean Niño 3.4 index was used. Warm (cold) events were identified whenever the standard deviation was greater (less) than 0.65 (−0.65) for at least 4 months (see Table S1). Among these events, ENSO occurrences were further classified as strong (weak) if the standard deviation of Niño 3.4 index’s absolute magnitude was higher than (less than) 1.3. Based on these definitions, strong and weak ENSO periods were identified (see Table S2). El Niño Modoki events were identified whenever the standardised EMI was at least 0.7^36^. Its corresponding one in the cold phase was less distinctive, probably due to the strong El Niño–La Niña asymmetry;^37^ hence, a La Niña Modoki was identified whenever the absolute value of the Niño 4 index was greater than that of the Niño 3 index within any La Niña period^38^. Finally, the composite method was used to extract recurrent climate signals accompanying ENSO occurrences. The Student’s t-test was used to assess the statistical significance of anomalies^39^.

## Additional Information

**How to cite this article**: Sohn, S.-J. *et al.* How do the strength and type of ENSO affect SST predictability in coupled models. *Sci. Rep.* **6**, 33790; doi: 10.1038/srep33790 (2016).

## Supplementary Material

Supplementary Information

## Figures and Tables

**Table 1:** Description of the five coupled climate models used in this study.

Country	Institute	Model	AGCM/Resolution	OGCM/Resolution	Ensemble Members	Hindcast Period
Canada	MSC	CCCma CGCM3	AGCM3/T63L31	OGCM4/1.41°lon × 0.94°lat L40	10	1981–2010
		CCCma CGCM4	AGCM4/T63L31		10	1981–2010
Italy	CMCC	CMCC-SPSv2	ECHAM5.3/T63L19	OPA8.2/ORCA2 grid L31	10	1981–2010
Korea	PNU	PNU	CCM3/T42L18	MOM3/0.7~2.8 lat L29	10	1979–2009
USA	NASA	GMAO	GEOS-5/288×181L72	MOM4/720 × 410 L40	9	1982–2012
