# Supplementary material for: How do the strength and type of ENSO affect SST predictability in coupled models
Source: Sci Rep. 2016 Sep 21;6:33790. doi: 10.1038/srep33790 (PMC5030669; doi:10.1038/srep33790)
Supplement: Supplementary Information [file srep33790-s1.doc]

Supplementary Information for

**How do the strength and type of ENSO affect SST predictability in coupled models?**

Soo-Jin Sohn^1,*^, Chi-Yung Tam^2^, and Hye-In Jeong^1^

^1^Climate Prediction Department, APEC Climate Center (APCC), Busan, Republic of Korea

^2^Earth System Science Programme, The Chinese University of Hong Kong, Hong Kong, China

**Supplementary Figures**


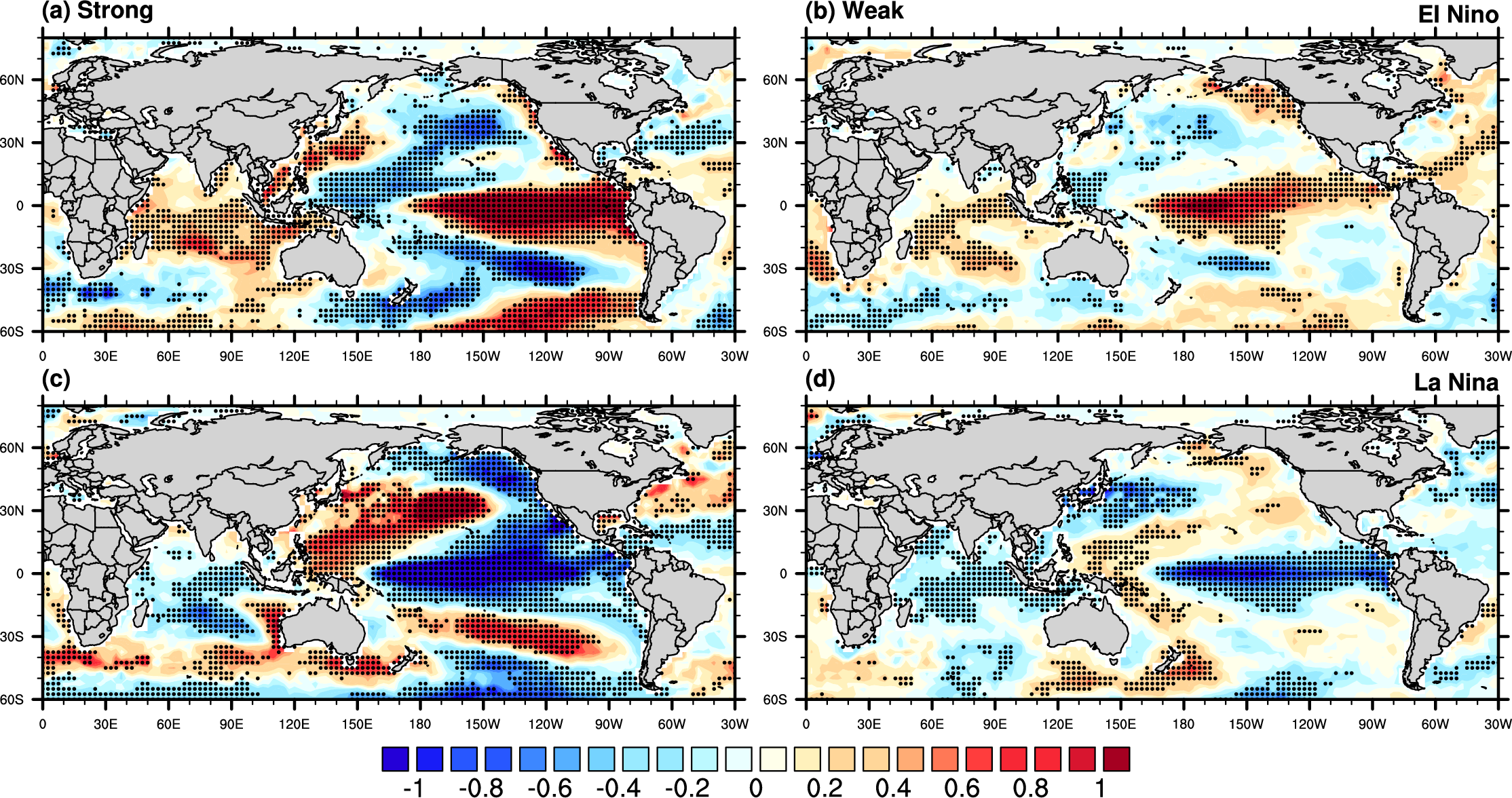


Figure S1. Composite maps of anomalous SST (shading) during (a, b) El Niño and (c, d) La Niña phases for (a, c) strong and (b, d) weak ENSO events during the boreal cold season. Black dots indicate grid points where the anomalies are statistically significant at the 95% level. These figures are generated by the NCAR Command Language (Version 6.3.0) [Software]. (2016). Boulder, Colorado: UCAR/NCAR/CISL/TDD. http://dx.doi.org/10.5065/D6WD3XH5.


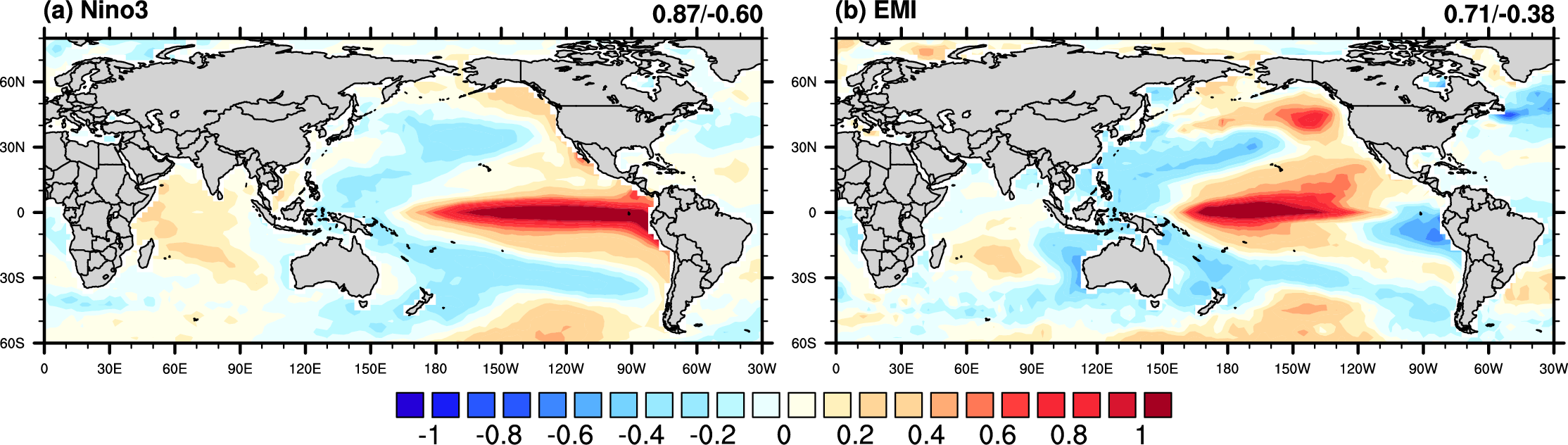


Figure S2. Regression coefficients of SST onto the (a) Niño3 index and (b) El Niño Modoki index (EMI) based on observations for all seasons during 1982-2006. Pattern correlation coefficients between the regression map based on the Niño 3 index (EMI) and strong El Niño/La Niña composites (weak El Niño/La Niña composites) during the boreal warm season are presented in the upper right of the left (right) panel. These figures are generated by the NCAR Command Language (Version 6.3.0) [Software]. (2016). Boulder, Colorado: UCAR/NCAR/CISL/TDD. http://dx.doi.org/10.5065/D6WD3XH5.


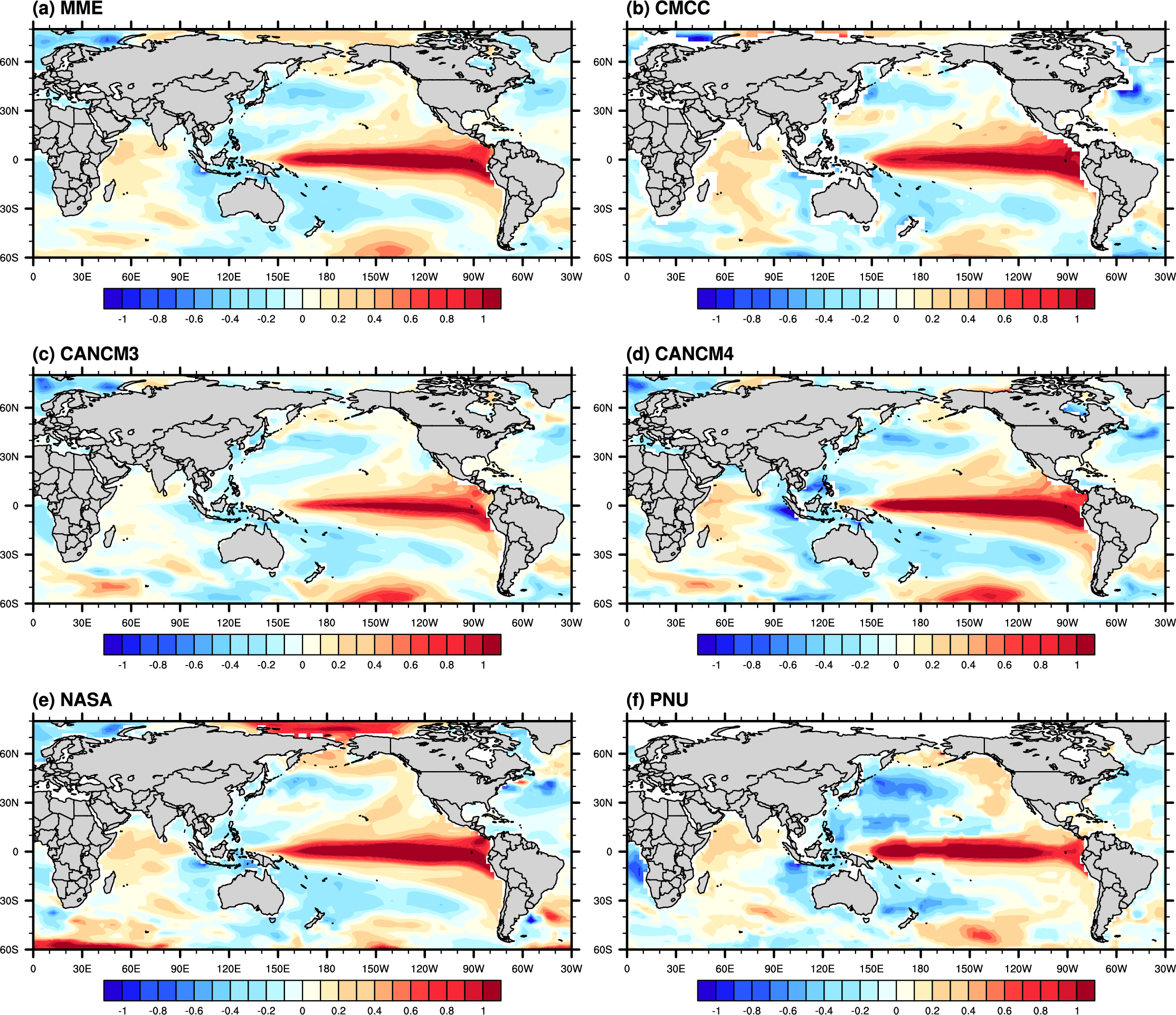


Figure S3. Composite maps of anomalous SST (shading) for strong El Niño events during the boreal warm season based on (a) MME mean and individual model simulations from (b) CMCC, (c) CANCM3, (d) CANCM4, (e) NASA and (f) PNU. These figures are generated by the NCAR Command Language (Version 6.3.0) [Software]. (2016). Boulder, Colorado: UCAR/NCAR/CISL/TDD. http://dx.doi.org/10.5065/D6WD3XH5.


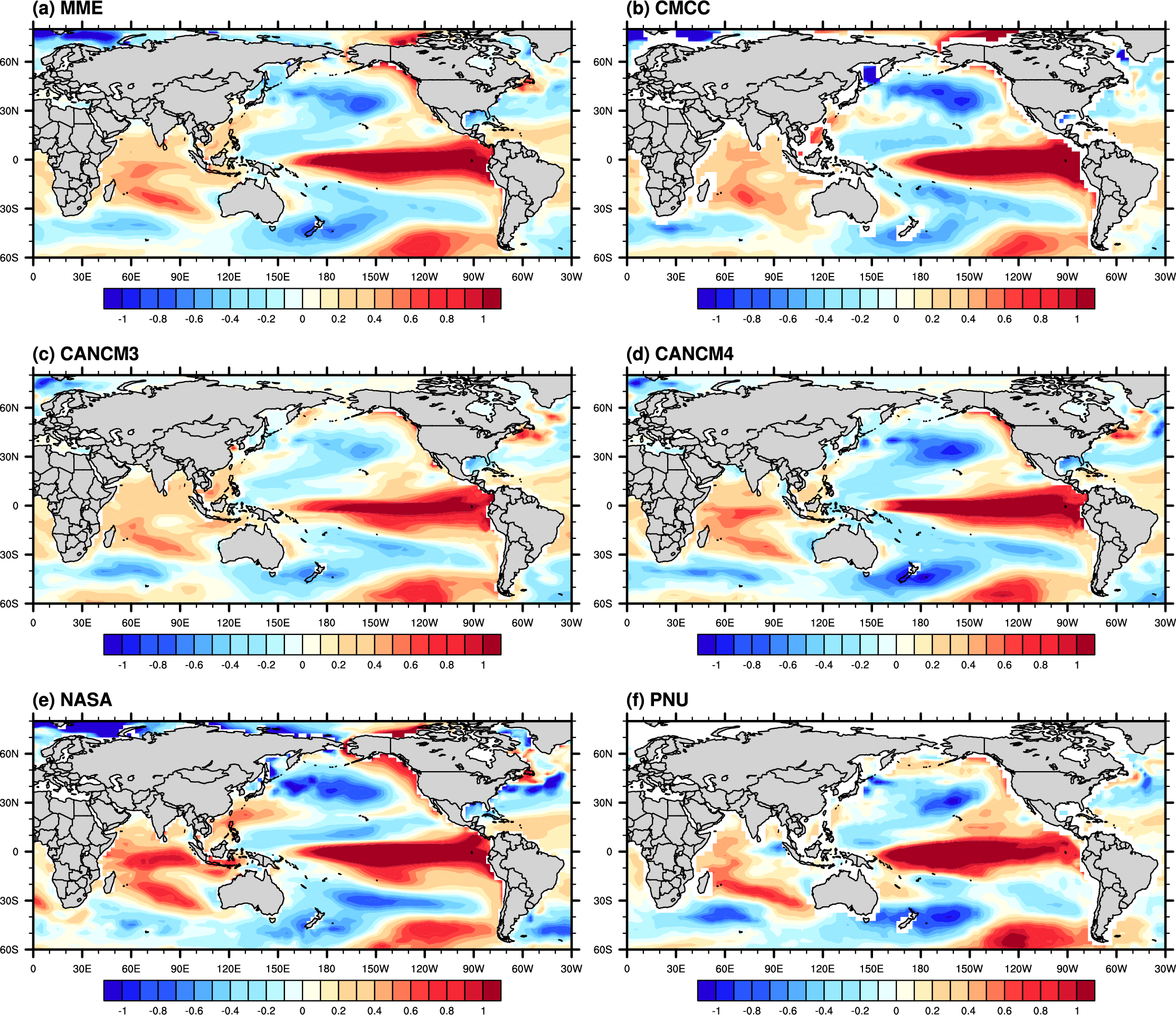


Figure S4. Composite maps of anomalous SST (shading) during strong El Niño in the boreal cold season based on (a) MME mean and simulations from (b) CMCC, (c) CANCM3, (d) CANCM4, (e) NASA and (f) PNU. These figures are generated by the NCAR Command Language (Version 6.3.0) [Software]. (2016). Boulder, Colorado: UCAR/NCAR/CISL/TDD. http://dx.doi.org/10.5065/D6WD3XH5.


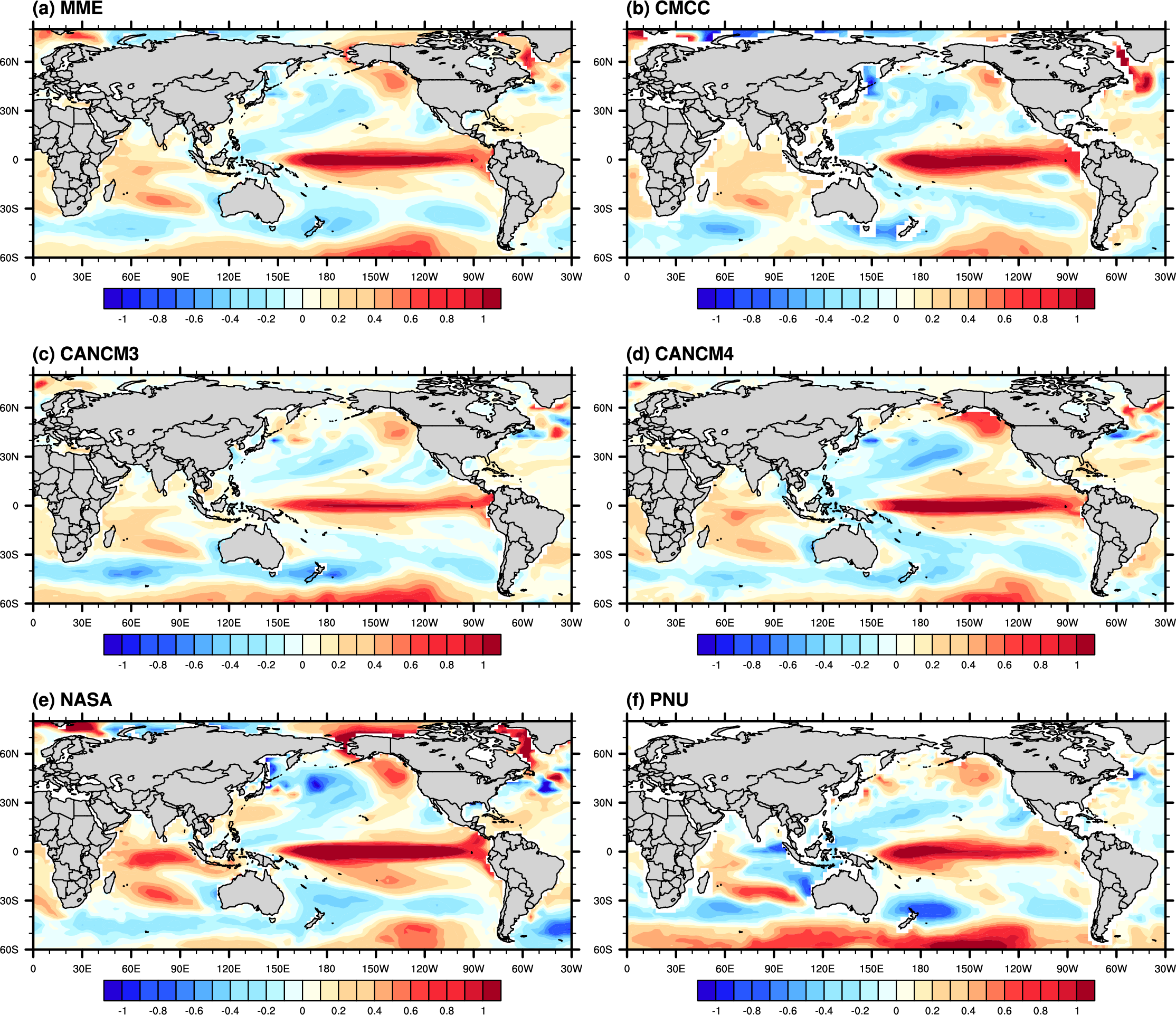


Figure S5. Composite maps of anomalous SST (shading) during weak El Niño in the boreal cold season based on (a) MME mean and simulations from (b) CMCC, (c) CANCM3, (d) CANCM4, (e) NASA and (f) PNU. There figures are generated by the NCAR Command Language (Version 6.3.0) [Software]. (2016). Boulder, Colorado: UCAR/NCAR/CISL/TDD. http://dx.doi.org/10.5065/D6WD3XH5.


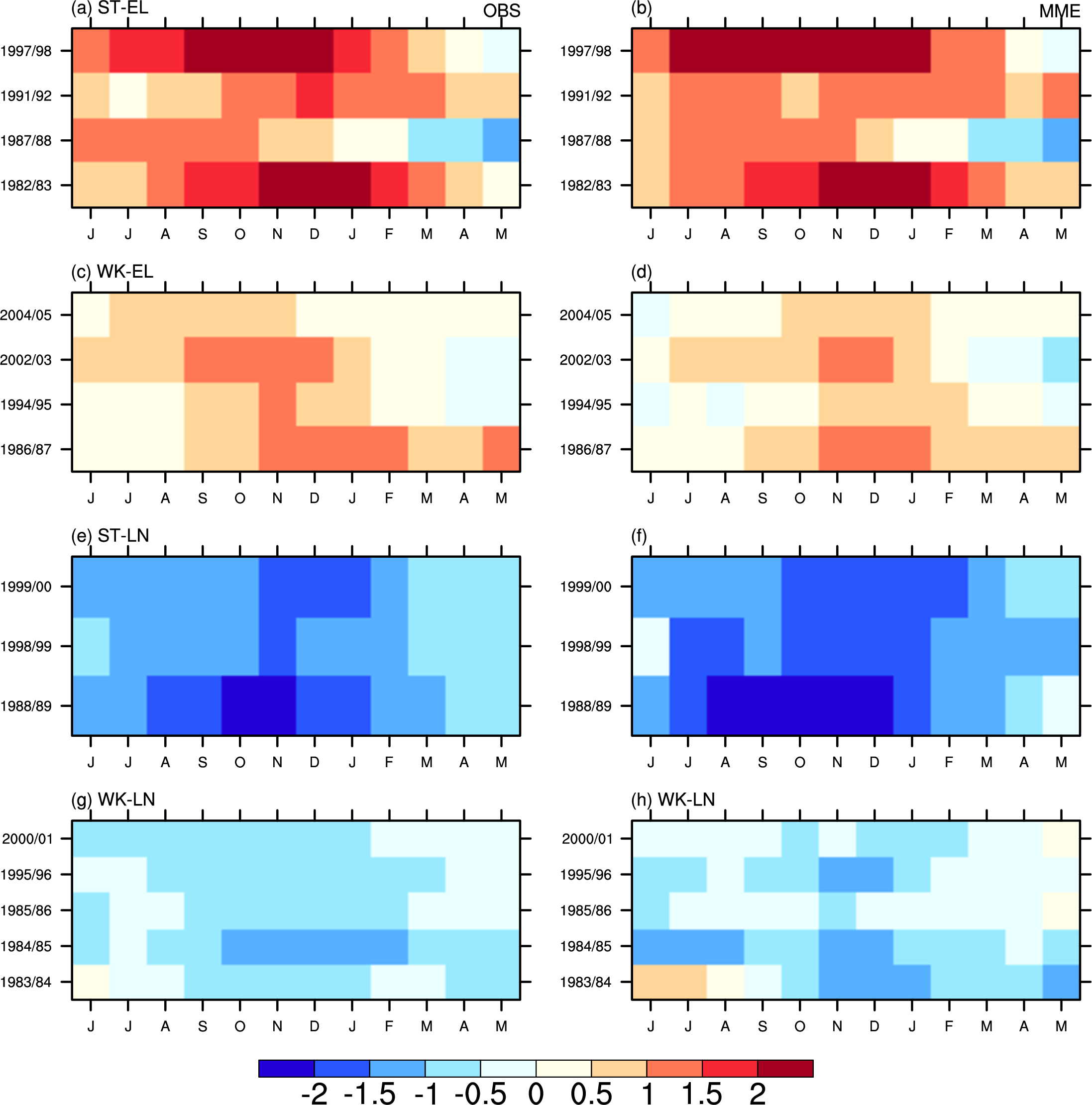


Figure S6. Evolution of the observed (left panels) and MME mean (right panels) forecasted Niño 3.4 index for (a, b) strong El Niño, (c, d) weak El Niño, (e, f) strong La Niña, and (g, h) weak La Niña events. These figures are generated by the NCAR Command Language (Version 6.3.0) [Software]. (2016). Boulder, Colorado: UCAR/NCAR/CISL/TDD. http://dx.doi.org/10.5065/D6WD3XH5.


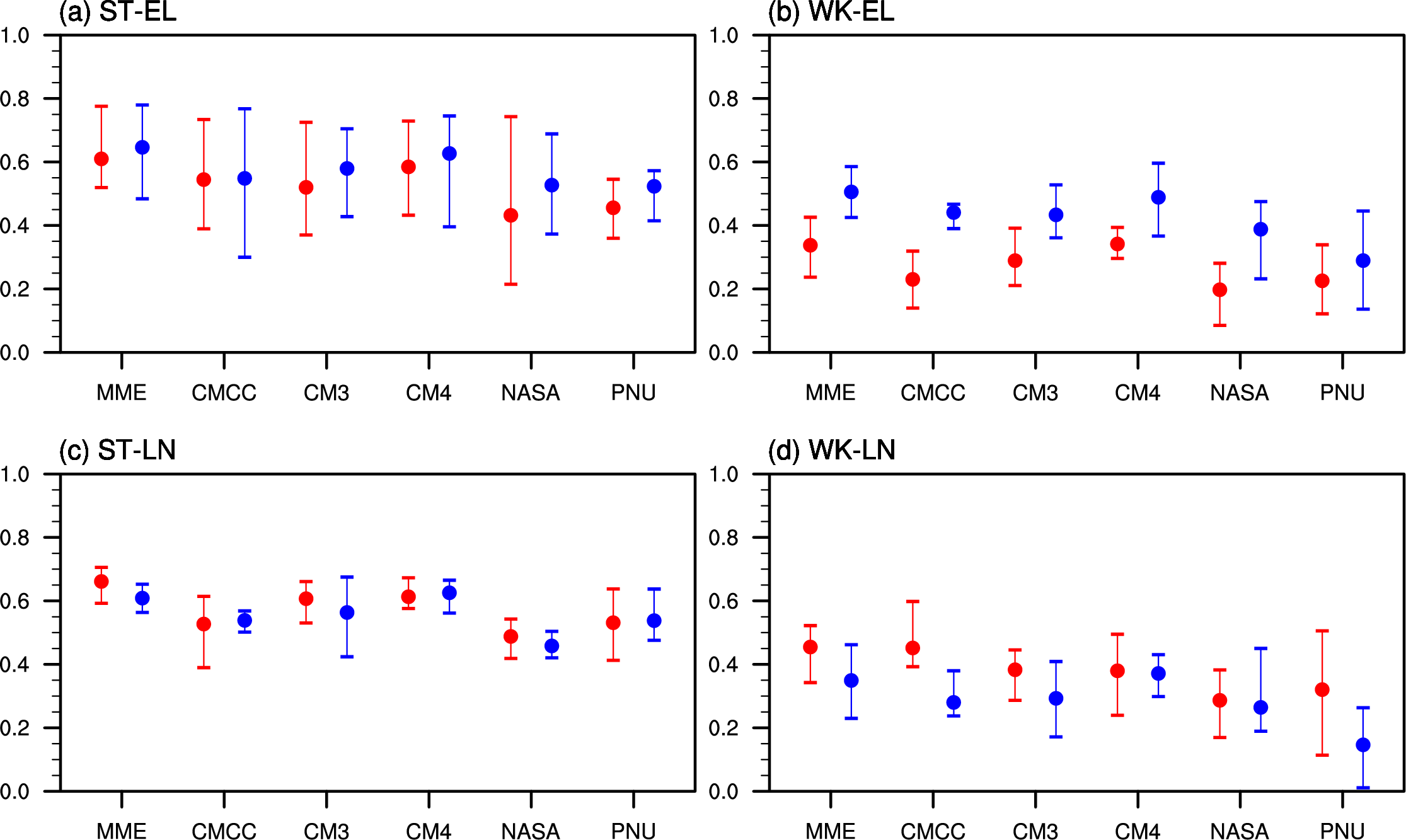


Figure S7. Mean (solid dot) and the range (bar) of anomaly pattern correlation coefficients (ACCs) between observed and model simulated SST patterns, computed based on data from (a) strong El Niño, (b) weak El Niño, (c) strong La Niña, and (d) weak La Niña years. Red and blue values denote statistics for warm and cold seasons, respectively. These figures are generated by the NCAR Command Language (Version 6.3.0) [Software]. (2016). Boulder, Colorado: UCAR/NCAR/CISL/TDD. http://dx.doi.org/10.5065/D6WD3XH5.

**
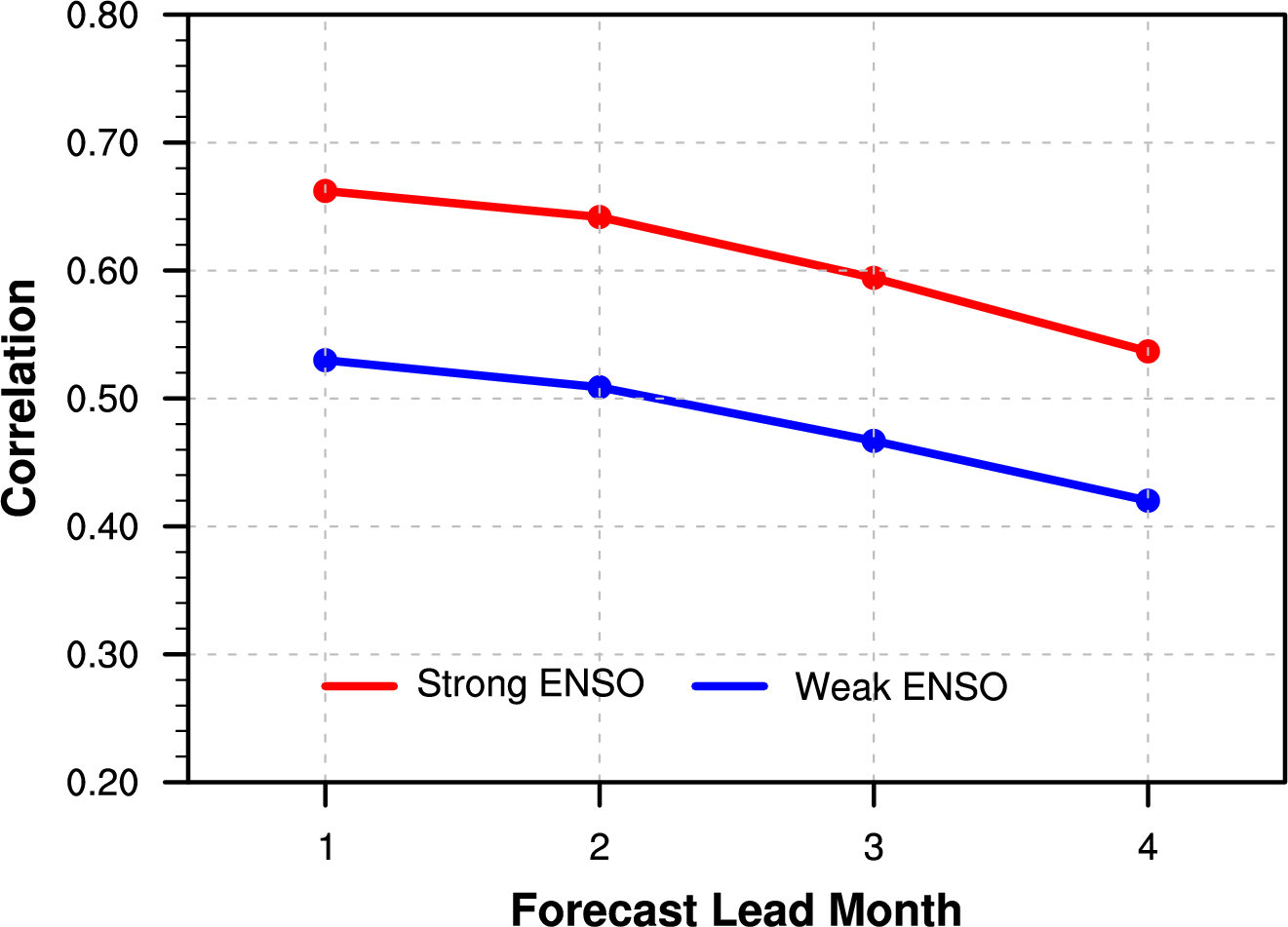
**

Figure S8. Anomaly pattern correlation coefficients (ACCs) between observed and MME mean forecasted SST patterns, computed based on data for strong ENSO (red line) and weak ENSO (blue line) events, as a function of forecast lead time. This figure is generated by the NCAR Command Language (Version 6.3.0) [Software]. (2016). Boulder, Colorado: UCAR/NCAR/CISL/TDD. http://dx.doi.org/10.5065/D6WD3XH5.

**Supplementary Tables**

Table S1. Classification of ENSO events.

Table S2. El Niño and La Niña events. Asterisks * and double asterisks ** correspond to El Niño Modoki and La Niña Modoki events, respectively. See text for details.

| El Niño | | La Niña | |
| --- | --- | --- | --- |
| Strong | Weak | Strong | Weak |
| 1982-1983 | 1986-1987* | 1988-1989** | 1983-1984** |
| 1987-1988 | 1994-1995* | 1998-1999** | 1984-1985 |
| 1991-1992* | 2002-2003* | 1999-2000 | 1985-1986 |
| 1997-1998 | 2004-2005* |  | 1995-1996 |
|  |  |  | 2000-2001** |
| 4 cases | 4 cases | 3 cases | 5 cases |
